# A Case of Morphine and Opium Habit Cured

**Published:** 1896-06

**Authors:** J. H. Powell

**Affiliations:** Higgston, Ga.


### A CASE OF MORPHINE AND OPIUM HABIT CURED.

J. H. POWELL, M. D., HIGGSTON, GA.

Mrs. C——, aged forty-six, a confirmed morphine, opium and chloral eater of twelve years standing, had been treated by several specialists only to fall back into the habit worse than ever, applied to me for treatment five months ago. She was very much emaciated, nervous, no appetite, continual diarrhœa for weeks past, stomach revolted all food, worse than crazy, unless taking from twenty to thirty grains of morphine during the day and night or its equivalent in chloral or opium. I never saw a more typical case of opium habit. My experience had been that the habit could not be removed in these confirmed cases, except where great will power is exercised on the part of the patient, and a doctor who never gives up as long as the patient will swallow his drugs, a thing they generally will do (fortunately).

But unless you can keep them under your immediate control, nearly everyone will slip in a little morphine with your medicine and put you under false impressions, as lying becomes second nature with all confirmed opium eaters. Knowing the patient as well as I did I refused to treat her. I was certain that I would fail to make a cure, and worst of all, be aggravated all my leisure time by her, as you are all aware how troublesome this class of patients are to deal with. I told her that I knew no more about treating such cases than the average physician.

She swallowed five grains of morphine in my presence and said she was done with doctors, anyway. Arriving home an hour afterwards she took a handful of chloral and soon became insensible. I was called, found her raving, cold and helpless. I used the stomach pump, afterwards washing out the stomach several times, gave three grains of atropine and a pint of brandy hypodermically in the course of twelve hours. In this time she had regained consciousness. Within twenty-four hours from the time I was called she was able to sit up. Her husband with tears begged me to treat her, and if I failed it would be no worse than the others had done. I concluded to try her "on my man," so to speak, and the results were very satisfactory. I gave her all the opium she wanted in pill form, allowing her to make the pills.

In preparing the opium for her I put in one-third gamboge; when it was mixed she could not detect it. It is needless to say she did not take very many of these pills. She soon became suspicious and bought some opium from a druggist. Her husband found it and I put gamboge in that; when she found her own opium had the same effect, she concluded I was right when I told her that she could not take opium with my medicine. She tried morphine, I managed to get apomorphia in that, she gave up in disgust and tried whiskey· A little wine of ipecac put in the whiskey soon broke her from that; she became discouraged and disgusted with the whole business and said she believed if she tried any of them again while taking my medicine it would kill her. In the meantime I was giving concentrated tinct. passiflora incarnata, as prepared by John B. Daniel, Atlanta, Ga., the best of all nervines in my estimation, and the best and most powerful calmative to nerve centers. I began the treatment with from six to ten teaspoonfuls daily, gradually dropping down to three, afterwards one at bedtime, continuing for some time, it gave perfect quiet, sweet and refreshing sleep. Within fifteen days my patient had a good appetite and bowels about regular, and seemed to be in normal condition. I gave the passiflora in all for five weeks, then wound up on nux vomica, muriatic acid and pepsin. Patient has become strong, plump and healthy, and is becoming a useful member of society.

I am sure she is entirely cured. I could enumerate several other cases treated in about the same way, but this one will suffice to show what passiflora will do in such cases. Let me urge every physician who is not using passiflora to try it especially in children and hysterical troubles of women, for nerve exhaustion, sleeplessness, convulsions, epilepsy, St. Vitus' dance. In hiccough, sleeplessness and tossing restlessness of low forms of fever I find it a specific.—From *California Medical Journal.*

---

PHENACETIN POISONING.—Dr. G. Kronig reports a fatal case. For occipital headache a seventeen-year old printer's assistant received five fifteen-grain phenacetin powders, not more than two to be used in a day. After an evening dose vomiting commenced, then we noticed great weakness and a bluish-gray coloration of face and lips. The temperature was 102.2 degrees F., the pupils were of medium size, the pulse weak, and the patient complained of headache, vomiting and diarrhœa. The urine was of a chocolate color, the conjunctivæ slightly jaundiced. General icterus followed and cynanosis of lips, hands and feet. Urine obtained by catheter was thick, dark reddish brown in color, containing masses of almost pure blood. Death followed two days after the ingestion of the remedy. As the patient was septic from a purulent otitis, a necropsy was necessary to show that death was due to the drug. The diagnosis reached by this means was stated as universal methæmoglobinæmia.—*Berlin Klinische Wochenschrift.*

